# Mining and Development of Novel SSR Markers Using Next Generation Sequencing (NGS) Data in Plants

**DOI:** 10.3390/molecules23020399

**Published:** 2018-02-13

**Authors:** Sima Taheri, Thohirah Lee Abdullah, Mohd Rafii Yusop, Mohamed Musa Hanafi, Mahbod Sahebi, Parisa Azizi, Redmond Ramin Shamshiri

**Affiliations:** 1 Department of Crop Science, Faculty of Agriculture, Universiti Putra Malaysia, 43400 Serdang, Selangor, Malaysia; mrafii@upm.edu.my; 2 Laboratory of Climate-Smart Food Crop Production, Institute of Tropical Agriculture and Food Security, Universiti Putra Malaysia, 43400 Serdang, Selangor, Malaysia; mmhanafi@upm.edu.my (M.M.H.); mahbod_sahebi@yahoo.com (M.S.); bahar3236@yahoo.com (P.A.); 3 Laboratory of Plantation Science and Technology, Institute of Plantation Studies, Universiti Putra Malaysia, 43400 Serdang, Selangor, Malaysia; 4 Department of Land Management, Faculty of Agriculture, Universiti Putra Malaysia, 43400 Serdang, Selangor, Malaysia; 5 Smart Farming Technology Research Center, Department of Biological and Agricultural Engineering, Faculty of Engineering, Universiti Putra Malaysia, 43400, Serdang, Selangor, Malaysia; raminshamshiri@upm.edu.my

**Keywords:** SSR markers, de novo transcriptome, RNA-Seq, microsatellite, Illumina, short tandem repeat (STR)

## Abstract

Microsatellites, or simple sequence repeats (SSRs), are one of the most informative and multi-purpose genetic markers exploited in plant functional genomics. However, the discovery of SSRs and development using traditional methods are laborious, time-consuming, and costly. Recently, the availability of high-throughput sequencing technologies has enabled researchers to identify a substantial number of microsatellites at less cost and effort than traditional approaches. Illumina is a noteworthy transcriptome sequencing technology that is currently used in SSR marker development. Although 454 pyrosequencing datasets can be used for SSR development, this type of sequencing is no longer supported. This review aims to present an overview of the next generation sequencing, with a focus on the efficient use of de novo transcriptome sequencing (RNA-Seq) and related tools for mining and development of microsatellites in plants.

## 1. Introduction

Advances in sequencing technologies, commonly referred to as next-generation sequencing (NGS), generate millions of sequences that can be read in a very cost-effective manner. NGS has paved the way for the large-scale discovery of genetic markers [1].

Within breeding programs, various types of molecular markers, such as random amplified polymorphic DNA (RAPD), ribosomal DNA (rDNA), inter-simple sequence repeat (ISSR), sequence characterised amplified region (SCAR), and simple sequence repeat (SSR), have been utilized [2,3,4,5,6,7]. Notably, SSRs and single nucleotide polymorphism (SNP) markers are propounded in genetic and plant breeding applications [8]. Furthermore, the advent of NGS has facilitated the development of SSRs or microsatellites across the genome, while being quick, efficient, and cost-effective even in non-model plant populations with limited or having any background genetic information [9,10,11].

In recent years, generating transcriptome data through RNA sequencing have been successfully reported for SSR marker development in non-model plants with no reference genome as de novo sequencing [12]. Accordingly, microsatellite markers have several uses in marker-assisted selection (MAS), linkage mapping or quantitative trait loci (QTL) mapping, phylogenetic, positional cloning, genetic divergence appraisal, genotypic profiling, and so forth [13,14].

The following discussion aims to review the application of next generation sequencing technologies specifically de novo transcriptome sequencing (RNA-Seq) in mining and development of SSR markers for genetic research.

### 1.1. Importance of Microsatellites and Their Use as Genetic Markers

Microsatellites are a subcategory of tandem repeats consisting of 1–6 nucleotides in length (motifs) found in genomes of all prokaryotes and eukaryotes [15]. Among individual genotypes, the number of repeat units may vary since the tandem arrays of SSR motifs change. Accordingly, with additional repeated units, the genotypic variety also increases. Likewise, motif length also affects the number of repeats as shorter motifs contain a higher number of repeats than larger (e.g., tetranucleotide) motifs. Notwithstanding, in smaller motifs, there is a greater feasibility of genotyping errors due to slipped-strand mispairing (stuttering) during the polymerase chain reaction (PCR), while longer and perfect SSR loci display more prominent allelic fluctuation [16,17].

There are a vast number of SSR loci spread out all over the genome, specifically in the euchromatin of eukaryotes, and in coding and non-coding nuclear and organellar DNA [18]. In a comparative study of rice and *Arabidopsis thaliana*, SSR distribution has been shown to be highly organised, varying in different regions of the genes [19]. Microsatellites have been utilized liberally over previous years since they are profoundly informative with a high mutation rate per locus per generation (10^−7^ to 10^−3^) [16], locus specificity, high intraspecific polymorphism, high reproducibility, ease of scoring, multiallelic, and frequent transpacific presence across related taxa. Additionally, the co-dominance nature of SSRs allows for the direct measurement of heterozygosity and only requires small amounts of DNA for data collection, another characteristic of SSRs (1 ng of DNA per reaction) [20,21,22,23]. Notably, they have been widely applied for different purposes, such as (1) genetic diversity; (2) discovering quantitative trait loci (QTL); (3) linkage map construction between gene and marker; (4) marker assisted selection for desired traits (MAS); (5) forensics and parentage analysis (SSRs with core repeats three to five nucleotides long are preferred); (6) cultivar DNA fingerprinting [24]; (7) genome-wide association study (GWAS); (8) gene flow estimation and crossing over rates; (9) marker assisted breeding (MAS) [25]; (10) haplotype determination; (11) harnessing heterosis; (12) germplasm characterization; and (13) genetic diagnostics, characterization of transformants, and the study of genome organization [14,26,27,28,29]. However, the high cost for SSR development, the presence of more null alleles, and the occurrence of homoplasy are some of the weak points of microsatellites [30].

SSRs are assorted based on their source, i.e., genomic SSRs (g-SSRs) and expressed sequence tags SSRs (EST-SSRs), which are located in the coding region and are identified from transcribed RNA sequences [31]. The EST-SSRs generate higher quality patterns with almost 70% having a distinct polymorphic fragment of the supposed size [32] as opposed to 36% in g-SSRs [33]. Furthermore, generating SSR markers using express sequence tags (EST) has been accelerated through sequencing technology advancements in various plant species [34,35,36,37,38]. Some characteristics of EST-SSRs such as their inexpensive development, a higher level of genetic diversity, and higher transferability to related taxa, are because of the additional conservation of sequences that contain EST-SSRs, thereby making them advantageous for biodiversity studies [39]. In contrast to the EST-SSRs, genomic SSRs have less interspecific transferability because of the repeat region or degeneracy of the primer binding sites [40,41]. Although a major weak point of the EST-SSRs is the sequence redundancy that yields multiple sets of markers at the same locus, this problem can be handled by assembling the ESTs into a unigene [41]. Accordingly, EST-SSRs markers have been developed and used in many plant species, such as rice, wheat, barley, sorghum, tomato, coffee, rubber, castor bean, and sesame [42,43,44,45,46,47,48,49,50,51].

### 1.2. Next-Generation Sequencing (NGS)

Since its commercial availability in 2005, next-generation sequencing (NGS) technology has assisted researchers in recent years, providing excellent opportunities for life sciences [52]. Before NGS, the development process of SSRs was labor-intensive, economically costly, and time-consuming due to the necessity of building up genomic libraries for targeted SSR motifs in creating recombinant DNA molecules using restriction enzymes for DNA fragmentation. Additionally, the cloning of DNA fragments into a vector was performed, as well as sequencing of clones carrying SSRs [11,53,54]. Secondly, one of the most significant impediments to primer design for PCR in the validation of SSR markers procedures was the necessity of background information of genome sequences containing SSR repeats [55,56,57]. Thirdly, successful SSR development relied strongly on the amplification of the target locus by a primer designed from a single SSR locus to generate obvious polymorphism [55]. High-throughput NGS technologies as a powerful, quick, cost-effective, and reliable tool, transformed the field of discovery and development of molecular markers by generating an enormous amount of sequence data [58,59,60,61].

There are different NGS technologies such as 454 Roche (http://www.my454.com) as the first commercially NGS platform that was utilized, mostly for bacterial and viral genomes. Next, there is the Illumina genome analyzer (http://www.Illumina.com) used for complex genomes (human, plant, and mouse), ABI SOLID (http://www.thermofisher.com/my/en/home/life-science/sequencing/next-generation-sequencing/solid-next-generation-sequencing.html/), Pacific Bioscience (http://www.pacb.com/), Ion Torrent (http://www.thermofisher.com/us/en/home/life-science/sequencing/next-generation-sequencing.html/), Oxford Nanopore (http://www.nanoporetech.com), and Qiagen GeneReader (http://www.genereaderngs.com/) [62,63]. In all, these NGS technologies are applied for different uses, such as for multiplex-PCR products, whole genome sequencing, de novo assembly sequencing, RNA-Seq, somatic mutation detection, methylation detection, validation of point mutations, and metagenomics [63,64]. Currently, sequencing by synthesis (e.g., Illumina) is the most widely utilized NGS platform for SSR marker development [11,29,65]. Although the 454-pyrosequencing dataset is still being used in some laboratories, it is mostly being phased out and will soon be redundant.

Illumina technology has been upgraded in recent years, revolutionizing NGS by establishing the HiSeq series (2500/3000/4000) sequencing system. The latest Illumina HiSeq 4000 sequencing system with patterned one or two flow-cells, can produce up to 100 million reads per sample. Moreover, it has a reading length of 50/75/150 bp for data yields of 210–250 Gb, 650–750 Gb, and 1300–1500 Gb per flow cell in less than 3.5 days’ runtime, and with an accuracy greater than 99%, as compared to the original HiSeq and MiSeq systems (www.illumina.com). Furthermore, only Illumina can generate paired-end sequencing reads leading to high-quality sequence data due to enhancing the possibility of the alignment of the reference genome. Moreover, Illumina facilitates the detection of genomic Indels, inversions, novel transcripts, and genes. Moreover, in de novo sequencing, it can produce longer contigs by filling the gaps in the consensus sequence [66,67]. Every laboratory using the HiSeq 3000/HiSeq 4000 Systems can access the latest sequencing technology and increase their genomics power.

### 1.3. SSR Discovery by Transcriptome Sequencing (RNA-Seq)

SSR development can be reliant on either genomic DNA sequences or double-stranded DNA synthesised from single-strand RNA (cDNA) depending on the project objectives, the future research scheme, and the researcher’s ability to manage output data [68]. Although direct sequencing using DNA instead of RNA is more straightforward, as it does not require library construction and normalization, sequence assembly, annotation, and integration of unigenes [69,70,71,72,73], transcriptome sequencing (RNA-Seq) as a successful and effective approach can be used for transcriptome profiling, gene expression analysis, and the detection of functional genes [74,75]. Furthermore, it is usable for SSR mining, especially for plants without a reference genome (de novo assembly) [76,77,78]. Moreover, high reproducibility and few systematic differences among technical replicates make RNA-Seq data more profitable [79]. Even in non-model organisms with no reference genome, large amounts of expressed sequence data can be obtained using RNA-Seq technology [80,81], where the generated readouts of billions of bases each day from a solitary instrument can be utilized in the development of high throughput EST-SSRs [82]. Accordingly, this speeds up transcriptomes assembly, allowing for the identification of expressed genes including gene isoforms and gene products to be completed accurately and extensively [83,84,85,86,87,88,89]. In RNA-Seq, in the presence of a reference genome, the output reads align to a reference genome or to reference transcripts, while in the absence of reference genome or transcriptome information, it is required to map a genome-scale transcription comprised of both the transcript structure and the level of expression for each gene at any specific developmental stage [90,91,92,93]. As de novo transcriptome assembly functions independently from existing genomic sequences, it can be particularly useful for the analysis of non-model species containing large nuclear genomes, such as polyploids [85].

Transcriptome sequencing is an efficient way to generate superior resources for the vast discovery and development of SSR loci in plants and has provided an improved understanding of them (see Table 1). In a recent study, researchers developed SSR in Guar (*Cyamopsis tetragonoloba*, L. Taub.) using Illumina HiSeq 2000 technology and found 5773 SSR loci from 62,146 non-redundant unigenes. In this study, 20 primer pairs were designed and synthesised, with a total of 13 primer pairs successfully amplified in two target guar varieties, M-83 and RGC-1066. Amplification failure in the other seven SSR markers was attributed to the possibility of flanking primers extending across a splice site with a large intron or chimeric cDNA contigs [8,94]. In a study by Wei et al. (2016) [80], they identified 9933 EST-SSR markers among 39,298 unigenes in colored calla lily (*Zantedeschia rehmannii* Engl.) using an Illumina HiSeq 2000 instrument. Accordingly, out of 200 designed primer pairs, 58 were polymorphic among 21 accessions of colored calla lily [80]. In 2012, Li and colleagues performed another example using de novo transcriptome sequencing for providing EST datasets used for the development of SSR molecular markers. In that study, a total of 39,257 EST-SSRs from the rubber tree were identified using data generated by Illumina HiSeq 2000 [49]. RNA-Seq as a simple, straightforward, and reliable approach has been applied for EST-SSR development in many other species such as sesame [51], sweet potato [95], carrot [96], bamboo [97], peanut [98], pea [99], common bean [100], mungbean (*Vigna radiata*) [101], and *Hemarthria* species [89] (see Table 1).

## 2. Overview of the Process of SSR Development through Transcriptome de Novo Assembly Using the Illumina Platform

**Figure 1:**
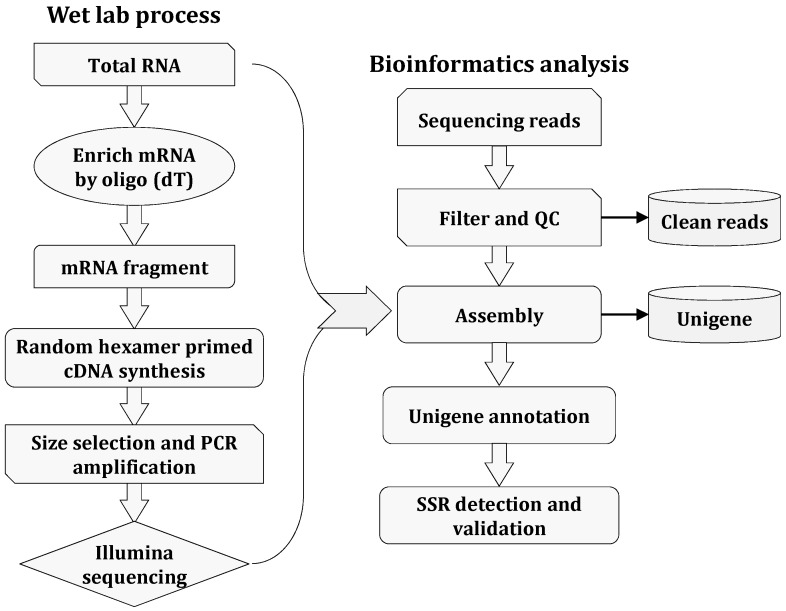
Schematic overview of a de novo transcriptome sequencing and assembly process.

The transcriptome de novo assembly process includes RNA extraction, cDNA library construction, sequencing, data filtering and quality control, de novo assembly, unigene annotation, SSR search and primer design, and marker validation (see Figure 1). After extraction of total RNA and its treatment with DNase I, Oligo(dT) is used to isolate mRNA. mRNAs are fragmented by fragmentation buffer and are used as a template for cDNA synthesis. Then, short fragments are purified and resolved with elution buffer (EB) for end reparation and single nucleotide A (adenine) addition. Next, adaptors are conjoined to short fragments, and suitable fragments are selected for PCR amplification. After quantification and qualification of the sample library during the QC steps, the library is then sequenced using an Illumina HiSeq 2000/2500/3000/4000, or another sequencer if necessary. After sequencing, the low-quality, adaptor-polluted, and high content of unknown base (N) reads will be filtered to obtain clean reads and are then saved in the FASTQ format [136]. Next, de novo assembly is performed with the clean reads to obtain the unigenes.

### 2.1. de Novo Assembly

There are several tools used for de novo assembly of RNA-Seq reads, such as Multiple-k [137], Rnnotator [138], Trans-ABySS [139], Velvet-Oases [140], and SOAPdenovo-Trans (http://soap.genomics.org.cn/SOAPdenovo-Trans.html). A tool that has recently been gaining popularity for de novo assembly of transcriptomes is Trinity [141,142], which generates individual de Bruijn graphs for sequence reads. Accordingly, each de Bruijn graph indicates the transcriptional complexity of a certain gene or locus, which is processed separately to obtain full-length splicing isoforms and to tease apart transcripts extracted from paralogous genes. Moreover, this process distinguishes Trinity from other available transcriptome de novo assembly tools. Additionally, Trinity sequentially applies three software applications, namely, Inchworm, Chrysalis, and Butterfly, to manage the enormous quantity of reads [138,143]. The process is briefly described below:

1. **Inchworm:** assembles the reads set into the unique sequences of transcripts by extending the sequences with the most abundant k-mers and then only reports the unique portions of differently spliced transcripts.
2. **Chrysalis:** groups the overlapping Inchworm contigs by overlaps of k − 1 into clusters to construct de Bruijn graph components for each cluster, representing the full transcriptional complexity of a given gene or genes with the common sequence. Next, chrysalis partitions the full read set between clusters.
3. **Butterfly:** resolves spliced and paralogous transcripts independently in parallel, ultimately reporting full-length transcripts.

**Figure 2:**
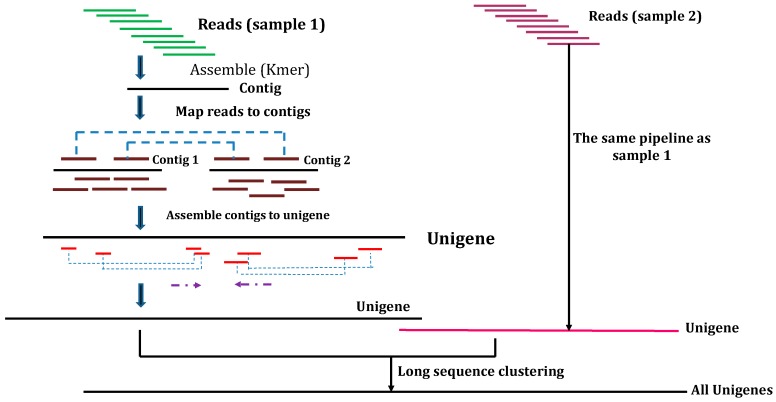
Schematic overview of the de novo transcriptome assembly process.

The transcripts generated by Trinity are applied to gene family clustering with the TGICL (TIGR Gene Indices clustering tools) pipeline [144]. Moreover, to obtain the final unigenes (if there is more than one sample), TGICL will execute again with each sample’s unigene to attain the final unigene (for downstream analyses). The unigenes will be divided into (a) clusters containing several clusters with more than 70% similarity and (b) singletons. Figure 2 illustrates the schematic overview of the process.

### 2.2. Unigene Functional Annotation

The functional databases used include the non-redundant nucleotide sequence database (NT), and the non-redundant protein sequence database (NR) of the National Centre for Biotechnology Information (NCBI), (http://www.ncbi.nlm.nih.gov). Additionally, the Swiss-Prot protein, Protein family (Pfam), Eukaryotic Orthologous Groups of proteins (KOG), Gene Ontology (GO), and the Kyoto Encyclopaedia of Genes and Genomes (KEGG). All databases are used to align assembled unigenes using Blast [145,146,147] (https://blast.ncbi.nlm.nih.gov/Blast.cgi) to obtain the annotated functions of each unigene. With the NR annotation, gene ontology annotations of the unigenes can be acquired using Blast2GO [148] or AmiGO [149]. The Gene Ontology (GO) project is a major bioinformatics collaboration to address the need of knowledge for descriptions of encoding biological functions by genes at the molecular, cellular, and tissue system levels across databases (http://www.geneontology.org).

### 2.3. Microsatellites Mining and Identification Tools

For SSR mining and identification in unigenes, tools such as MISA (MIcroSAtellite; http://pgrc.ipk-gatersleben.de/misa) [45,150] and SSR Locator [151] have been developed. However, these tools are not able to process large genomes efficiently and produce poor statistics. Additionally, as a platform-dependent tool, MISA does not provide a graphical interface or SSR Locator. The development of the Genome-wide Microsatellite Analysing Tool (GMATo) overcomes the abovementioned weak points, given it is faster and more accurate than MISA and SSR Locator. Furthermore, GMATo is an appropriate, powerful tool for complete SSR characterization in any genome size [152]. Recently, a novel software package, GMATA, was developed that provides new strategies and comprehensive solutions for fast SSR analyses, marker development, and polymorphism screening by mapping and graphically, displaying the results in a genome browser with other genic features. Furthermore, this software also provides high-quality statistical graphics to incorporate in publications [153]. Notably, GMATA is the first tool that generates results that enable viewing SSR loci and SSR marker information along with other genome features in a genome browser. Current software/tools, such as SSR Locator cannot easily design primers that flank each SSR locus in a large genome sequence because the genome sequence at the chromosome level is too large to be directly used as a template for primer design, as for large genomes, primer design can be quite difficult. The GMATA software only uses the flanking sequence as a template for designing PCR primers, thereby reducing computing memory and accelerates the design process for large data sequences. Furthermore, not all primer pairs are unique at the genome scale because duplicated DNA sequences have arisen during evolution. The mining of SSRs from the whole genome provides valuable information on the abundance of SSRs in various genomic regions and will also facilitate the development of markers for genetic analysis and related applications, such as marker-assisted breeding and linkage mapping [154]. Additionally, the Whole Genome Sequencing (WGS)-SSR Annotation Tool (WGSSAT) provides a graphical user interface (GUI) pipeline, mining and characterizing SSR from whole genome data.

The sequences will be searched for perfect mono-, di-, tri-, tetra-, penta-, and hexanucleotide motifs. Based on previous studies, dinucleotide and trinucleotide repeat motifs are the most frequent SSR repeats in *Hemarthria* species [89], *Dipteronia Oliver* [108], *Amorphophallus* [31], and pigeon pea [72]. Mono-nucleotide repeats will be excluded since they can result from sequencing errors or mismatches. Furthermore, distinguishing mononucleotides from polyadenylation might be difficult. From the unigenes, primers can then be designed using Primer 3 (http://bioinfo.ut.ee/primer3) [155], or Premier 5.0 (PREMIER Biosoft International, Palo Alto, CA, USA), or similar software. Designing primers should meet some criteria, such as the size of the PCR product range between 100 and 280/300 bp; a primer length of 18–21/28 nucleotides; a GC content of 40–70% with 50% as the optimum, and with an annealing temperature between 50 and 70 °C, with 55 °C as the optimum melting temperature [31,108].

### 2.4. DNA Isolation, PCR Amplification, and SSR Validation

In order to validate the SSRs, the DNA will need to be isolated from plant leaves. DNA integrity will be checked by gel electrophoresis (1% agarose gel). Accordingly, all designed SSR primers should be tested for amplification in different plant varieties or accessions through polymerase chain reaction (PCR). The successful primers will then be selected for genetic diversity studies.

### 2.5. Genotyping STRs in Next-Generation Data: Challenges and Solutions

Short tandem repeats (STRs) or microsatellites are highly variable elements that play a crucial role in population genetics applications as molecular markers [156]. However, there is a limitation on genotyping STRs from high-throughput sequencing data (for a review, see Treangen and Salzberg, 2012) [157]. From a bioinformatics perspective, if whole reads carrying STRs are mapped due to high mismatch/indel resulting from different STR lengths, some reads will not be mapped with those at the corresponding positions in the reference genome. This leads to a much less accurate estimation of the allele frequency and the real level of STR variation in the genome [158]. More recently, a number of software tools have been developed to profile STRs in NGS data, such as LobSTR [159], RepeatSeq [160], STRViper [161], STR-FM [158], PSR [162], rAmpSeq [163], and STRScan [164]. LobSTR has a fast running time and considers PCR stutter noise during the genotyping stage. However, LobSTR sensitivity is low for mononucleotide STRs and STRs shorter than 25 bp. Additionally, LobSTR uses a mapping algorithm that is fixed in the program [157]. Therefore, an STR-profiling tool was needed to customize a mapping algorithm that can evaluate and correct the STR errors generated by NGS technology [154].

The RepeatSeq tool was released using informed error profiles from inbred *Drosophila* lines [160]. The tool utilizes the reads mapped by other programs, such as Burrows-Wheeler Aligner (BWA) [165] and Bowtie [166], and predicts the most probable genotype at a locus based on the STR motif, length, and base quality. However, RepeatSeq’s limitation is in using the whole-read mapping approach, which introduces a bias toward the STR length in the reference genome and thus might obscure the true STR variation spectrum. To profile the full spectrum of STR lengths in human and other genomes, and to correct for NGS-associated STR errors, STR-FM (short tandem repeat profiling using a flank-based mapping approach) was developed as a flexible pipeline for detecting and genotyping STRs from short-read sequencing data. Moreover, this pipeline can detect STRs of any length, including short ones (as short as only two repeats), and includes an error-correcting module, which can combine any NGS mapping algorithm with paired-end mapping capability, thereby making it adaptable to new mapping methods as they become available [158].

Another method that exploits paired-end information for the detection of STR variation from in-depth sequencing data is STRViper [161]. STRViper predicts the polymorphic repeats across a population of genomes and uncovers several polymorphic repeats including the locus of the only known repeat expansion in *A. thaliana*. All tools require prealigned data, except lobSTR, which uses its own aligner. STRViper’s performance largely depends on the fragment size variance. Therefore, regarding running time, once reads were aligned, both lobSTR and RepeatSeq performances were poor on moderate variation sizes. Notably, STRViper needed <4 min to process 10-fold coverage reads [161].

All tools mentioned above are used mainly for profiling microsatellites from SAM/BAM data that they identify gSSR alleles at each locus in short reads NGS data. However, they have difficulties in the correct identification of polymorphic SSRs. Unlike the tools above, polymorphic SSR retrieval (PSR) was developed to identify polymorphic SSRs from NGS data where, in the non-model plant species, they use de novo transcriptome assembly as a first sequence resource for SSR mining more effectively [162]. In 2016, Buckler et al. [163] developed the rAmpSeq tool for repeat amplification sequencing that is applicable for genotyping in most species, using low-quality DNA and generating several markers, thereby facilitating whole genome sequencing at less cost per sample. In the last decade, genomics has been used in scientific discovery of thousands of species, but breeding or conservation applications were strongly felt for only a few dozen species. Another software tool, STRScan, was developed for in silico mining STRs from genome sequences with higher sensitivity compared to lobSTR and STR-FM. It uses a specific algorithm for targeted STR profiling in NGS data on the whole genome sequencing (WGS) data from both the Sanger sequencer [167] and the Illumina sequencer (generated by the 1000 Genomes Project [168]). The results showed that STRScan could profile 20% more STRs in the target set, which were missed by lobSTR, in less computation time.

## 3. Conclusions

Molecular markers are tools used to detect genetic polymorphism at specific loci and an entire genome level in plant species. Among the various molecular markers, SSRs are remarked as being among the most important in genetic and plant breeding programs. However, limited numbers of SSRs are known for some species, thus limiting the capacity of plant breeding approaches. The ability of next-generation sequencing accelerated microsatellite identification and facilitated their variation discovery. Presently, the utilization of RNA-Seq or transcriptome profiling as a reliable and robust tool brings interesting opportunities in the identification and development of a substantial number of SSR markers, being faster, easier, and more cost-effective compared to traditional SSR development processes. The RNA-Seq provides an extensive collection of transcriptomes (expressed sequences), which are believed to be more transmissible among tightly related species as compared to genomic markers because of their presence in more-conserved transcribed regions of the genome. Several studies on SSR development have demonstrated that Illumina is the most frequently used platform to generate millions of transcriptome sequences, which vary in length. Illumina HiSeq4000 has higher accuracy and is less expensive compared to Illumina HiSeq 2500/3000 sequencing and is the best platform to isolate EST-SSRs markers. Over the years, to support the management of vast amounts of NGS sequence data, and for the profiling and genotyping of short tandem repeats, new specific tools have been developed. Therefore, the utilization of NGS technologies in the development of SSRs is an effective method for the plant community, especially in non-model plants which no genetic information is known.

## Figures and Tables

**Table 1:** Developed simple sequence repeat (SSR) markers using Illumina, and 454 sequencing technologies in plants.

Species	SSR Type	No. Unigenes	NGS Technology	Total No. of Discovered SSRs	Total No. of SSR Primer Designed	Total Polymorphic SSR Primers	Reference
*Jatropha curcas*	SSR	115,611	Roche 454 Genome Sequencer	9798	262	33	[102]
Guar (*Cyamopsis tetragonoloba*, L. Taub.)	SSR	62,146	Illumina HiSeq 2000 sequencing platform	5773	20	13	[8]
Red clover (*Trifolium pratense* L.)	SSR	80,328/83,489/; 84,545/84,442	Illumina HiSeq 2000 sequencing platform	15	n/a	15	[103]
Winged bean (*Psophocarpus tetragonolobus*)	EST-SSR	97,241	Roche 454 Genome Sequencer FLX (Titanium chemistry)	12,956	2994	n/a	[104]
Colored calla lily (*Zantedeschia rehmannii* Engl.)	EST-SSR	39,298	Illumina HiSeq 2000 sequencing platform	9933	200	58	[80]
*Salix psammophila*	EST-SSR	71,458	Illumina HiSeq2500 platform	6346	168	27	[105]
Sainfoin (*Onobrychis viciifolia*)	SSR	92,772	Illumina Hiseq 2000 sequencing platform	3823	100	n/a	[106]
Two *Hemarthria* Species	SSR	137,142/77,150	Illumina HiSeqTM 2500 sequencing platform	10,888	4846	34	[89]
Oak (*Quercus austrocochinchinensis*) & (*Q. kerrii*)	SSR	49,845/50,767	Illumina MiSeq sequencing platform	13,762/13,430	5196/5021	18	[107]
*Dipteronia oliver* (Aceraceae)	SSR	99,358	Illumina Hiseq 2000 sequencing platform	12,377	4179	97	[108]
*Elymus sibiricus* L.	EST-SSR	94,458	Illumina HiSeq2000 sequencing platform	8769	500	112	[109]
*Argyranthemum broussonetii,* *Echium wildpretii*, *Descurainia bourgaeana*	SSR	80,620	Illumina MiSeq sequencing platform	2282	30	8	[110]
		58,526		1284	n/a	n/a	
		44,287		1972	n/a	n/a	
*Boea clarkeana* Hemsl. (*Boea, Gesneriaceae*)	EST-SSR	91,449	Illumina HiSeqTM 2000 sequencing platform	8563	436	17	[111]
Diabelia (Caprifoliaceae)	EST-SSR	58669	Illumina HiSeqTM 2000 sequencing platform	n/a	2746	13	[112]
*Paris polyphylla* Smith	EST-SSR	56,095	Illumina HiSeq2000 sequencing platform	3853	80	9	[113]
Pummelo (*Citrus grandis* (L.) Osbeck)	SSR	57,212	Illumina HiSeq2000 sequencing platform	10,276	1174	29	[114]
Chinese walnut (*Juglans cathayensis* L.)	EST-SSR	116814	Illumina HiSeq2000 sequencing platform	22,484	62	12	[115]
Chinese cabbage (Brassica rapa L. ssp. pekinensis)	EST-SSR	51,694	Solexa/Illumina	10,420	24	17	[116]
Lotus (*Nelumbo nucifera*)	SSR	105,834	Illumina HiSeqTM 2000 sequencing platform	11,178	6568	80	[117]
*Carthamus tinctorius* L. (Safflower)	SSR	2,043,956	Illumina HiSeqTM 2000 sequencing platform	23,067	325	93	[118]
*Phalaenopsis aphrodite* subsp. formosana	EST-SSR	22,598	Illumina HiSeqTM 2000 sequencing platform	1439	1051	10	[119]
*Neolitsea sericea* (Lauraceae)	EST-SSR	68,624	Illumina HiSeqTM 2000 sequencing platform	13,213	1191	13	[120]
Mango (*Mangifera indica*)	SSR	66,288	Illumina HiSeq 2000 sequencing platform	106,049	84,118	90	[121]
Adzuki bean (*Vigna angularis*)	EST-SSR	112 million	Illumina HiSeq2000 sequencing platform	7947	296	38	[41]
*Quercus pubescens*	SSR	96,006	Illumina HiSeq 2000 sequencing platform	14,202	10,864	20	[122]
*Brassica oleracea* L. var. *capitate* L.	EST-SSR	34,688 and 40,947	454 GS FLX Titanium Sequencer	2405	937	116	[123]
*Hevea brasiliensis*	SSR	19,708	Roche 454 sequencing platform	1397	n/a	n/a	[124]
*Medicago sativa*	EST-SSR	54,278	Illumina HiSeqTM 2000 sequencing platform	4493	837	372	[125]
*Paspalum dilatatum* Poir.	EST-SSR	20169	GS FLX Titanium technology	2339	96	32	[126]
Red clover (*Trifolium pratense* L.)	SSR	45181	Illumina HiSeq2000 sequencing platform	3127	2193	n/a	[76]
*Eulaliopsis binata*	SSR	59,134	Illumina HiSeq 2000 sequencing platform	6681	5,723	24	[127]
Common vetch (*Vicia sativa* subsp. *sativa*)	cDNA-SSR (cSSR)	n/a	454 Pyrosequencing platform	3811	300	65	[128]
Faba bean (*Vicia faba* L.)	cDNA-SSR (cSSR)	n/a	454 Pyrosequencing platform	1729	240	55	[129]
lentil (*Lens culinaris* Medik.)	SSR	55,463	Illumina Genome Analyzer II platform	8722	5,673	23	[130]
*Amorphophallus* (Araceae)	SSR	135,822	Illumina HiSeq™ 2000 sequencing platform	19,596	10,754	205	[31]
*Tea (Camellia sinensis)*	SSR	75,531	Illumina HiSeq™ 2000 platform	12,582	2439	431	[131]
Faba bean (*Vicia faba* L.)	cDNA-SSR (cSSR)	n/a	454 Pyrosequencing platform	1729	240	55	[129]
Tea (*Camellia sinensis*)	EST-SSR	25,637	Roche/454 Genome Sequencer FLX Instrument	3767	100	36	[132]
Rubber tree (*Hevea brasiliensis* Muell. Arg.)	EST-SSR	22,756	Illumina HiSeqTM 2000 sequencing platform	39,257	110	61	[49]
Peanut (*Arachis hypogaea* L.)	SSR	59,077	Solexa HiSeq™ 2000 sequencing platform	3919	160	65	[79]
*Bituminaria bituminosa*	SSR	3838	Roche 454 sequencing platform	3419	240	21	[133]
(*Sesamum indicum* L.)	EST-SSR	86,222	Illumina HiSeq2000 sequencing platform	7702	50	40	[51]
Pigeonpea [*Cajanus cajan* (L.) Millspaugh]	SSR	43,324	454 GS-FLX sequencing platform	3771	2877	20	[72]
Chickpea (*Cicer arietinum* L.)	SSR and SNP	103,215	Roche⁄454 and Illumina⁄Solexa	26,252	3172	42	[71]
Lentil (*Lens culinaris* Medik.)	EST-SSR	25,592	Roche 454 GS-FLX Titanium platform	1.38 × 10^6^	2393	51	[134]
*Hevea brasiliensis*	EST-SSR	113,313	454 pyrosequencing platform	17,819	430	47	[135]
Sweet potato (*Ipomoea batatas*)	cDNA SSR (cSSR)	56,516	Illumina paired-end sequencing platform	4114	100	92	[86]
